# Novel Allelic Gene Variations in *CmCLAVATA3* (*CmCLV3*) Were Identified in a Genetic Population of Melon (*Cucumis melo* L.)

**DOI:** 10.3390/ijms25116011

**Published:** 2024-05-30

**Authors:** Hangyu Wu, Yue Jia, Xinxiu Chen, Naiyu Jiang, Zhonghua Zhang, Sen Chai

**Affiliations:** Engineering Laboratory of Genetic Improvement of Horticultural Crops of Shandong Province, College of Horticulture, Qingdao Agricultural University, Qingdao 266109, China; whyu688@163.com (H.W.); jiayue@qau.edu.cn (Y.J.); cxxlddx@163.com (X.C.); jny980312@163.com (N.J.); zhangzhonghua_79@163.com (Z.Z.)

**Keywords:** melon, carpels develop, map-base cloning, genetic selection

## Abstract

Carpel number (CN) is an important trait affecting the fruit size and shape of melon, which plays a crucial role in determining the overall appearance and market value. A unique non-synonymous single nucleotide polymorphism (SNP) in *CmCLAVATA3* (*CmCLV3*) is responsible for the variation of CN in *C. melo* ssp. *agrestis* (hereafter *agrestis*), but it has been unclear in *C. melo* ssp. *melo* (hereafter *melo*). In this study, one major locus controlling the polymorphism of 5-CN (multi-CN) and 3-CN (normal-CN) in *melo* was identified using bulked segregant analysis (BSA-seq). This locus was then fine-mapped to an interval of 1.8 Mb on chromosome 12 using a segregating population containing 1451 progeny. *CmCLV3* is still present in the candidate region. A new allele of *CmCLV3*, which contains five other nucleotide polymorphisms, including a non-synonymous SNP in coding sequence (CDS), except the SNP reported in *agrestis*, was identified in *melo*. A cis-trans test confirmed that the candidate gene, *CmCLV3*, contributes to the variation of CNs in *melo*. The qRT-PCR results indicate that there is no significant difference in the expression level of *CmCLV3* in the apical stem between the multi-CN plants and the normal-CN plants. Overall, this study provides a genetic resource for melon fruit development research and molecular breeding. Additionally, it suggests that *melo* has undergone similar genetic selection but evolved into an independent allele.

## 1. Introduction

Carpels are crucial female reproductive organs in angiosperms and play a key role in the evolution and adaptation of flowering plants [1,2]. Carpels not only protect ovules from microbial and insect damage, but also provide an essential space for plant fertilization and fruit development [3]. Furthermore, most of our food originates coming from carpel tissues, making this of significant importance for the yield traits of horticultural crops [4,5]. As a unique structure of the pistil, carpels form the ovary. During this process, their inner walls generate septa, dividing the ovary into different locules [4]. The variation in the number of these carpels (or locules) can influence the development of flower morphology and fruits, thereby causing an effect on fruit shape [5]. In nature, the number of carpels in most plants is relatively stable due to genetic regulation [6]. However, a small portion of plants may undergo variations in carpel development, leading to changes in the number of carpels. Furthermore, changes in the number of carpels can influence the shape and size of the fruit. For instance, cultivated tomatoes with ten or more locules are larger than the wild types with two [7]. The *Xishuangbanna* cucumber, with five carpels, has spherical fruits, unlike typical cucumbers with three carpels that produce elongated fruits. These examples show that carpel number can influence fruit shape [8,9,10]. 

The homeostasis of the shoot apical meristem (SAM) has a significant impact on the development of carpels [11]; abnormal development of the SAM can lead to disruption in the proliferation and differentiation of floral organs. Plants with multiple carpels typically have larger SAMs, underscoring the critical influence of SAM development on carpel phenotype [12]. The core of SAM homeostasis are controlled by the *CLAVATA3* (*CLV3*)–*WUS*CHEL (*WUS*) pathway [11]. Within the SAM development, the CLV3 peptide plays a critical role in regulating stem cell proliferation and differentiation [13]. This secreted peptide inhibits the WUS transcription factor critical for stem cell niche maintenance [14,15], impacting plant growth and development [12,16,17]. The interaction between WUS and CLV3 is essential [18], involving receptor groups CLV1, CLV2, and CORYNE (CRN), which collectively regulate SAM size and floral organ development [19,20,21]. The *CLV3*–*WUS* pathway, coordinated by multiple receptors and co-receptors, establishes a crucial negative feedback loop for maintaining SAM size and development [12]. Indeed, the number of carpels in many crops is currently related to the function of the *CLV3*–*WUS* pathway. In tomatoes (*Solanum lycopersicum* L.), the number of locules (chambers) is associated with two QTLs, *fw2.1*(*lc*) and *fw11.3*(*fas*), and the *fas* locus has an epistatic effect on the *lc* locus [8,22]. The *fas* locus covers a 1600 bp region on the promoter of *WUSCHEL*(*SLWUS*). Two SNPs in this region have a significant impact on the number of floral organs in modern tomato varieties [23]. When *SLWUS* is silenced, the number of locules in tomatoes decreases [24]. This evidence suggests a potential application for genetic modification and selective breeding in crop improvement strategies [7,23]. Current reports on the CNs in melons are also related to this pathway. A genome-wide association study (GWAS) of 297 diverse melon accessions identified a pleiotropic SNP^(G-C)^ on chromosome 12 within the gene *CmCLV3*, which affects CNs [25]. BSA is widely used in plants to identify specific genes and genomic regions. Its analysis not only pinpoints trait-associated regions but also provides numerous molecular markers. For example, disease-resistant markers obtained through BSA analysis, like INDEL and SNP markers, aid in screening and breeding powdery mildew-resistant melon varieties [26,27]. Similarly, INDEL markers can verify cucumber seed purity [28]. These studies demonstrate the linkage of obtained intervals to phenotypes. Researchers used BSA and fine-mapping of the CN-related region of *agrestis* within an 80 kb region on chromosome 12. Within this region, the candidate gene *CmCLV3* was identified, containing a unique nonsynonymous SNP^(G-C, Pro-Arg)^ at position 15,486,217, which may be associated with CN in *agrestis* [29].

Melons are divided into two subspecies: *agrestis* and *melo*; the multi-CN materials used in the studies mentioned above were all from *agrestis* [30]. While candidate genes regulating carpel number have been pinpointed in *agrestis*, their counterparts in *melo* remain unstudied, and the regulatory mechanisms in melons are unclear. This study utilized a genetic cross between the multi-CN *melo* CR365 and the normal-CN variety W336. Using BSA and Kompetitive Allele Specific PCR (KASP) markers, we confined the relevant genomic region to a 1.8 Mb segment on chromosome 12. Allelic testing with multi-CN *agrestis* identified the candidate gene *CmCLV3*, with mutations differing from those in *melo*. Expression analysis using qRT-PCR showed no significant difference in candidate gene expression between extreme phenotypes. Our findings offer new insights into CN variation and provide molecular markers useful for melon breeding programs.

## 2. Results

### 2.1. Genetic and Phenotypic Analysis of Melon Carpel Number

**Figure 1:**
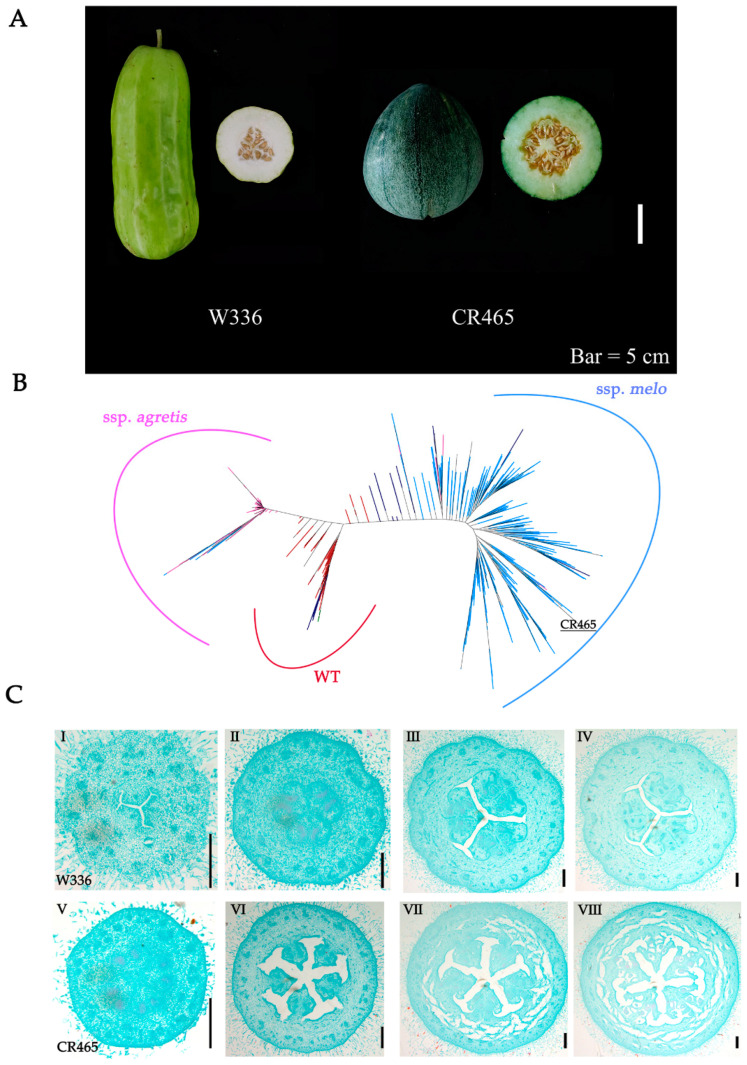
Variation in carpel number between CR465 with W336 and analysis of carpelloid phenotypes in *melo* varieties. (**A**) Cross-sectional representation of mature carpel numbers for W336 and CR465. Bar = 5 cm. (**B**) Phylogenetic tree of melon. Red represents wild-type materials, pink represents *agrestis*, and blue represents *melo*. (**C**) Paraffin sections of ovary diameters for W336 and CR465; (**I**–**IV**) represent the cross-sections of the ovary of W336 at 1 mm, 4 mm, 8 mm, and flowering stages, respectively, while (**V**–**VIII**) represent the cross-sections of the ovary of CR465 at 1 mm, 4 mm, 8 mm, and flowering stages, respectively. Bars = 0.4 mm.

We identified the melo CR465 and W336 from some melons collected in earlier stages of our research. CR465 and W336 exhibited multi-CN and normal-CN, respectively (Figure 1A). An evolutionary tree was constructed by combining CR465 with 1175 publicly available data [30]. Consistent with previous findings, the resulting delineated three major clades, with all wild accessions clustering together, and agrestis and melo forming distinct branches [30]. CR465 was situated within the melo branch, indicating closer genetic relatedness to this group (Figure 1B). Additionally, resequencing data and PCR analysis showed that there is no SNP in *CmCLAVATA3* (*CmCLV3*), as reported previously in agrestis between CR465 with W336 [30]. Therefore, the CR465 we have discovered represents a prospective multi-CN melo material.

To investigate the structural basis of carpel, we compared the carpel shape at different developmental stages between CR465 and W336 (Figure 1C). Carpel differentiation is discernible as early as the 1 mm stage. At the 4 mm stage, the carpel number is clearly visible, and at the 8 mm stage, as well as during the flowering period, the number of carpels is fixed, and the carpels are mature, with ovules formed. A comparison between CR465 and W336 shows distinct differences, indicating that the determination of carpel number occurs early in the development of the flower.

In order to understand the genetic mechanisms underlying CN in melon, we crossed the multi-CN CR365 with the normal-CN W336. The F_1_ progeny exhibited phenotypically normal-CN. Among the 183 F_2_ individual plants investigated, 142 plants had normal-CN and 41 plants had multi-CN, which is consistent with a single Mendelian trait segregating in a ratio of 3:1 (Table 1). Therefore, only one locus controls CN in this segregating population and multi-CN is a recessive character.

### 2.2. Localization of the Number of Melon Carpels Gene

**Figure 2:**
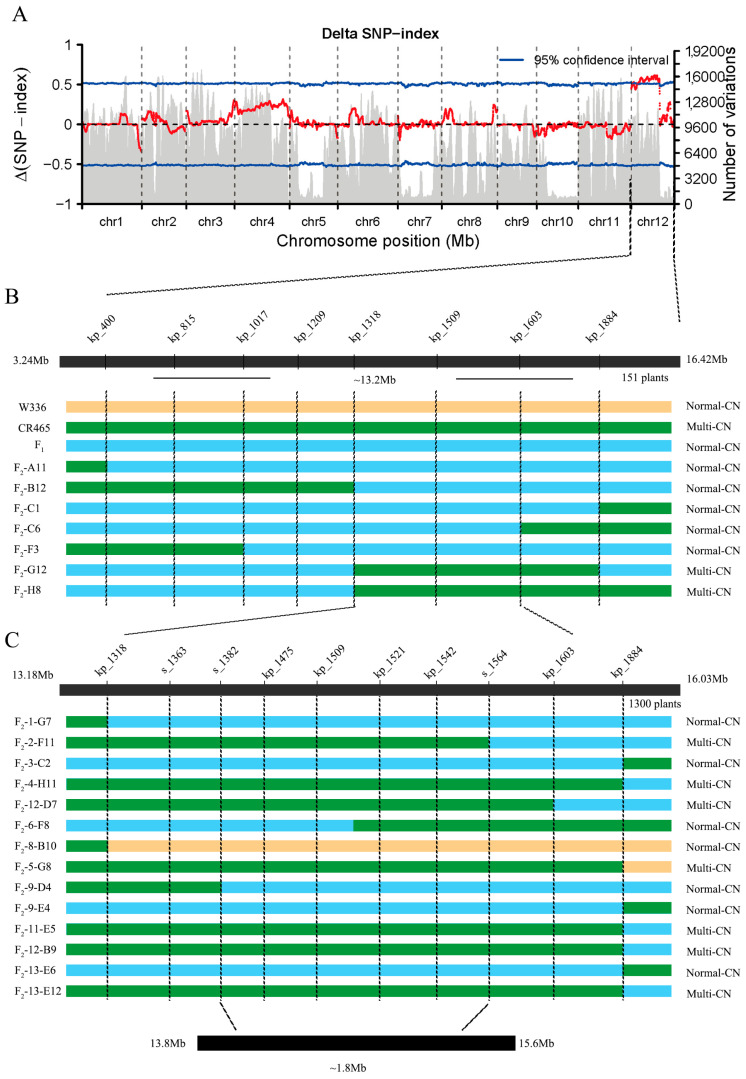
Map-based cloning of the gene controlling CN in *melon***.** (**A**) The *x*-axis represents chromosome names, the left *y*-axis denotes Delta SNP Index Values, and the right *y*-axis indicates the number of chromosomal variants. The blue line represents the 95% threshold value, and the red points show the distribution of SNP values across the chromosomes. (**B**) Primers used within the interval and recombinant individuals are shown, with material names on the left and phenotypes on the right, pale yellow represents normal-CN, green represents multi-CN, and blue represents heterozygous F_1_. (**C**) A detailed mapping diagram for the F_2_ population, with elements corresponding to those in figure (**B**).

We used the BSA-seq method to map the casual gene for the variation of CN in melon. We selected 18 individuals with normal-CN from the F_2_ segregating population as the ‘normal-CN pool’ and selected 18 individuals with multi-CN as the ‘multi-CN pool’. The two pools (normal-CN pool and multi-CN pool) were aligned to the reference genome (DHL92 v4.0) to obtain Variant Call Format (VCF) files containing single nucleotide polymorphisms (SNPs) and insertions-deletions (INDELs). Different homozygous variant sites in the two pools were identified, and the SNP/INDEL-index values for these sites were calculated. Finally, the differences in variations across all chromosomes between the two pools were computed to obtain the ΔSNP/INDEL-index values. Regions where these values exceeded the 95% confidence interval were identified as candidate intervals for the genes (Figure 2A). Analysis of the data revealed a prominent peak on chromosomes 12, ranging from 3.24 Mb to 16.42 Mb (*p* < 0.05). 

Subsequently, we developed two competitive allele-specific PCR (KASP) markers: chr12_4009797 (kp_400) and chr12_18842048 (kp_1884). Then, we used them to screen the 152 individuals in the segregating population and 14 recombinant individuals were selected. We continued to develop another seven KASP markers between kp_400 and kp_1184, and used them to screen the 14 recombinants. The gene was preliminarily mapped between markers kp_1318 and kp_1603 in an interval between 13.18 and 16.03 Mb (Figure 2B). To fine-map the gene, we performed high-resolution genotyping on a larger set of 1300 F_2_ plants in 2023. Six additional KASP markers ware developed to help us map the casual gene. Ultimately, the target gene was fine-mapped to the 1.8 Mb interval between maker s_1382 (13,827,296 bp) and s_1564 (15,643,145) bp on chromosome 12 (Figure 2C).

### 2.3. Identification of Candidate Genes and Study of Mutation Mechanism

**Figure 3:**
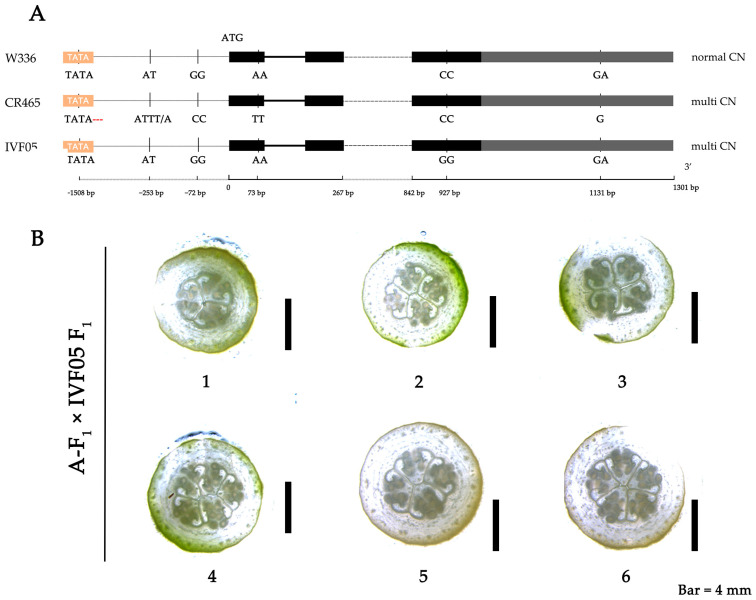
Mutations in *CmCLV3* across different materials and carpel number phenotypes in reciprocal test cross F_1_ progeny. (**A**) Black boxes represent the gene’s coding sequence (CDS) regions, gray boxes represent the 3′ untranslated region (3′ UTR), dotted lines denote length omissions, and variations before ATG are promoter mutations, with the sequence extending from 5′ UTR to 3′ UTR from left to right. (**B**) Carpel number phenotypes of different progeny from reciprocal cis-trans test, 1–6 represent A-F_1_ × IVF05 F_1_-1 to A-F_1_ × IVF05 F_1_-6.

Within the finely mapped interval, the gene *MELO3C035640 (CmCLV3)*, annotated as a *CLAVATA3* protein, is a candidate gene controlling CN in plant *melo.* Analysis of the VCF data within the *CmCLV3* region revealed five variants: three promoter region alterations (an INDEL^15,215,094^ resulting in a missing TATA box, an INDEL^15,213,839^ from AT to ATTT/A, and a SNP^15,213,658^ from G to C), a 3′ UTR variation (INDEL^15,212,455^ from GA to A), and a nonsynonymous mutation (SNP^15,213,513^ from A to T leading to a Phe to Ile change) (Figure 3A). The nonsynonymous mutation is unique compared to the SNP previously reported [30]. This mutation was confirmed in the parental CR465. To distinguish from the variant identified in previous research on *agrestis* varieties, we refer to the previously reported site within the gene as *CmCLV3^H1^* (*CmCLV3^H1^* = 15,212,659) and the newly identified site as *CmCLV3^H2^* (*CmCLV3^H2^* = 15,213,513). A KASP marker was designed for the *CmCLV3^H2^* site, which showed significant polymorphism. The *CmCLV3^H2^* site was homozygous A/A and homozygous T/T in W366 and CR465, respectively. Normal-CN individuals were either homozygous A/A or heterozygous A/T and multi-CN individuals were homozygous T/T at the *CmCLV3^H2^* site. This KASP marker cosegregated with the CN.

### 2.4. Cis-Trans Test for Polycarpous Candidate Genes

To determine whether the multi-CN phenotype in *melo* is caused by mutations in *CmCLV3*, we crossed F_1_ plants with normal carpel numbers (*CmCLV3^H1^* = C/C; *CmCLV3^H2^* = A/T, normal-CN) with the previously reported mutant material IVF05 (*CmCLV3^H1^* = G/G; *CmCLV3^H2^* = A/A; multi-CN). We then cloned and identified the two mutation sites in the progeny and analyzed them in conjunction with the phenotypic information of the hybrid offspring. The results showed that the genotype of the normal-CN plants was A/A, C/G, while the multi-CN plants had a genotype of A/T, C/G (Figure 3B). 

Upon the phenotypic assessment of numerous F_1_ progeny plants, a pronounced concordance between genotype and phenotype was discerned (Table 2), with the multi-CN trait in the F_1_ generation conforming to the expectations of the cis-trans test. This substantiates *CmCLV3* as a candidate gene responsible for the variation in CN, and the results aforementioned furnish compelling evidence supporting the validation of this candidate gene.

### 2.5. Analysis of the Expression Patterns of Candidate Genes and Prediction of Protein Structures

Since the differentiation of carpel number in melons is determined before flowering and *CmCLV3* specifically expressed in the shoot apex [26], we analyzed the expression pattern in lateral shoot apices. Due to the small size of individual shoot apices and their low expression levels, we collected and pooled lateral shoot apices from 15 normal-CN individuals and 15 multi-CN individuals in F_2_ population, respectively. We conducted qRT-PCR analysis on the candidate gene *CmCLV3* and its pathway-related gene *CmCLV1*(*MELO3C003743*); the results indicate that there is no significant difference in the relative expression levels of *CmCLV3* between multi-CN samples and normal-CN samples. Similarly, the expression levels of *CmCLV1* as a receptor for *CmCLV3* also show no significant difference. Therefore, the expression level of *CmCLV3* may indeed not be the main cause of CN variation.

**Figure 4:**
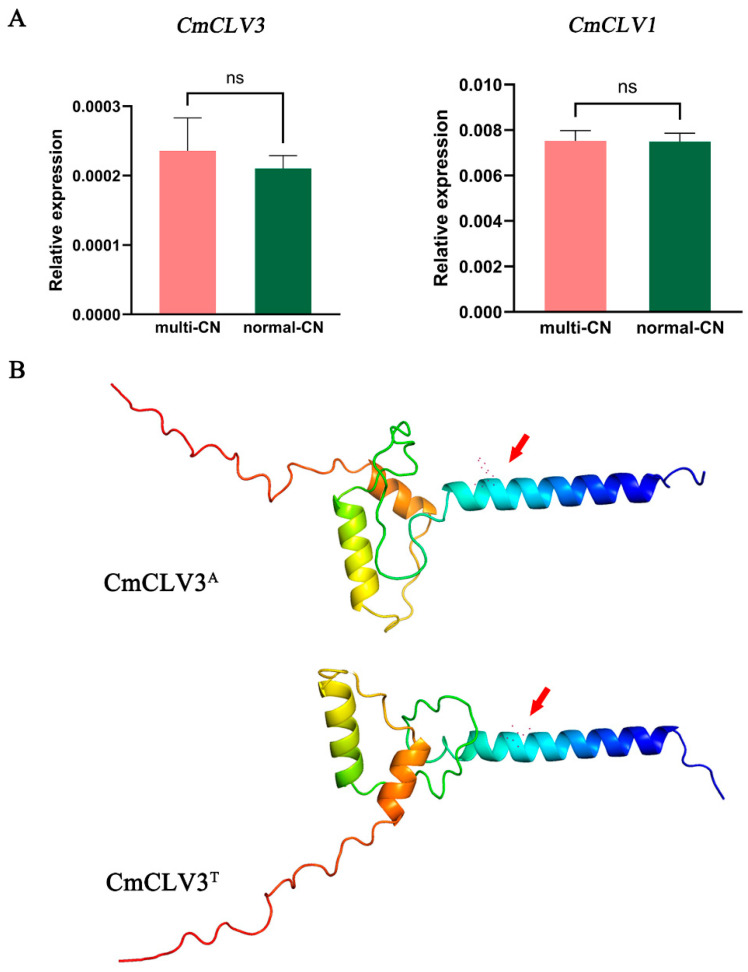
Analysis of the expression patterns and protein structure prediction of the candidate gene. (**A**) Analysis of the expression patterns of the candidate gene. The left figure represents the expression levels of *CmCLV3* and the right one represents the expression levels of *CmCLV1*. “ns” indicates no significant difference. (**B**) Protein structure prediction conducted by AlphaFold2 [31]. The red arrows point to the mutation sites, the bluer the color, the higher the prediction confidence.

In parallel, we employed software AlphaFold2 for structural predictions of both the wild-type and mutant proteins [31]. Our analysis revealed that in the multi-CN variant, the amino acid substitution from phenylalanine to isoleucine resulted in an increased number of protein helices, which in turn precipitated a conformational shift in the overall structure (Figure 4B). This structural modification could conceivably lead to an alteration in protein functionality.

## 3. Discussion

The CN is an important trait that affects the shape and size of melon fruits and has a significant impact on fruit yield and economic benefits. Exploring multi-CN melon resources and investigating the genetic mechanisms of CN in *melo* contribute to the improvement in melon varieties, market requirement, and the profitability of growers. *Agrestis* and *melo* are classified into different subspecies and represent separate branches of domestication. Melons were domesticated separately in India and Africa, exhibiting significant genetic and agronomic trait divergence. *Agrestis* were selected for traits such as flesh thickness and loss of bitterness, whereas *melo*, which are closer to wild types, show different selection patterns, such as in the *CmPH* gene related to fruit acidity. Genetic structure analysis revealed substantial differentiation between the subspecies, with a high FST index of 0.46, indicating pronounced population differences, a finding supported by further population studies in 2020 [25,30]. Hence, *agrestis* and *melo* exhibit substantial genetic divergence, indicating the likelihood of distinct mechanisms regulating CN.

To address this, our investigation leveraged a genetic cross between the multi-CN, *melo* CR465, and the normal-CN, elliptical melon W336, thereby generating a novel genetic population. This cross revealed that all F_1_ offspring presented with the normal-CN phenotype, and the F_2_ generation adhered to a 3:1 segregation ratio of normal to multi-CN, indicative of monogenic control of this trait, with the normal-CN phenotype being dominant. Advancements in sequencing have enhanced BSA’s efficiency in identifying genomic regions linked to heritable traits. Our study pinpointed the melon CN gene locus to a central region on chromosome 12 (3.24–16.42 MB). Further refinement with KASP markers and recombinant F_2_ plant analysis narrowed this to a 1.8 M span (13.8–15.6 MB). To ascertain whether the genetic determinants for CN in *agrestis* and *melo* were identical, we conducted reciprocal crosses using F_1_ plants and the *agrestis*, multi-CN melon IVF05. The resulting phenotypic and genotypic data revealed that *CmCLV3^H2^* in *melo* may be a crucial mutation causing multi-CN. This finding confirms that the *CmCLV3^H2^* mutation we identified is a recessive mutation located within the same gene as *CmCLV3^H1^*, reinforcing the candidacy of *CmCLV3* as a key gene in the regulation of CN in melons.

Although the cis-trans test validated the presence of mutations within the candidate gene, the distinct mutation sites observed in *agrestis* and *melo* intrigued us. In our *melo* materials, we identified mutation sites different from those in *agrestis*, a phenomenon that may result from independent differentiation under varying environmental conditions. The two subspecies may regulate CN through different divergent loci, a concept supported by similar research in multiple crops. For instance, loss-of-function (LoF) mutations in *Pdh1* are the primary genetic basis for pod shattering resistance in cultivated soybeans, with distinct haplotypes of this gene distributed differently between wild and cultivated soybeans. The distribution of *Pdh1* haplotypes in cultivated and landrace varieties correlates closely with precipitation levels, a pattern not observed in wild types, suggesting environmental influences on genetic differentiation within the gene across subspecies [32]. In strawberries, the loss of anthocyanin synthesis function leads to white strawberries, with *MYB10* being the key gene controlling this trait. A pan-genome study in strawberries revealed different *MYB10* mutation sites in various strawberry species, causing distinct amino acid changes in *MYB10*. This research indicates that multiple *MYB10* mutations may be a crucial factor in the transition from deep red and pink to completely white strawberries [33]. White and wild grapes share two SNP mutations in *VvMybA*, both of which cause the regulatory factor to lose the ability to synthesize anthocyanins, preventing the grapes from turning red [34,35]. The two mutations in *agrestis* and *melo* may both result in changes in protein function, altering the feedback regulatory capacity of *CmCLV3* on SAM, ultimately leading to the formation of multi-CN.

The distinct selection loci we have identified may also reflect the divergence of the two subspecies under environmental pressures. However, functional studies of these different alleles will require further experimental evidence to support their roles.

## 4. Materials and Methods

### 4.1. Plant Materials and Phenotypic Analysis

In this study, the multi-CN melon CR465 (P_1_) and the normal-CN melon W336 (P_2_) were used as parental lines. The previously established F_1_ generation was self-pollinated in spring 2022 to produce the F_2_ population. In autumn 2022, 200 F_2_ plants were grown for genetic analysis and BSA-seq. The following spring, 1300 F_2_ plants were cultivated for fine-mapping. In the summer of 2023, F_1_ plants were crossed with the multi-CN *agrestis* IVF05 to obtain F_1_ seeds, which were then planted in autumn for phenotypic observation and allelic testing. CN in melons was assessed by manually sectioning fruits from lateral branches 5 weeks after planting, followed by decolorization in 75% alcohol. After decolorization, the CNs was observed and recorded. For each plant, more than three ovaries were examined, and the average number of carpels per fruit was calculated. Plants with an average CN of less than 4 were considered normal-CN, while those with an average exceeding 4 were classified as multi-CN. Phenotypic data were recorded in Excel 2019 and analyzed using the CHISQ function to perform chi-square tests, determining whether the observed segregation ratios conformed to expected genetic patterns.

To observe changes in CNs at different stages in more detail, parental ovaries at 1 mm, 4 mm, and 8 mm stages were fixed in FAA, vacuum-infiltrated twice for 20 min, and stored at 4 °C for 24 h before being sent to Servicebio Company in Wuhan, China for paraffin sectioning. The sections were photographed under an optical microscope for observation. In the F_2_ generation, the CNs were counted using the manual sectioning method. First, the sections were decolorized in 75% alcohol, and then observed and counted under a microscope. The melon IVF05 we used was provided by Dr H. Wang from the Institute of Vegetables and Flowers (IVF), Chinese Academy of Agricultural Sciences. Other plant materials originated from the seed bank at Qingdao Agricultural University’s Vegetable Function Research Center and were cultivated in the greenhouses at the Agricultural Hi-Tech Industry Zone, Jimo, Shandong (36°56′ N, 120°21′ E).

### 4.2. Constructing a Phylogenetic Tree

We resequenced CR465 and mapped the reads to the genome DHL92 v4.0 [33]. We obtained a natural population of 1175 accessions genotype data from the publicly available Cucumis melo genome [32]. After merging and preliminary filtering 1175 data with CR465 data, 9,168,820 high-quality SNPs were obtained, from which 14,915 fourfold degenerate codon transversion SNPs (4DTV SNPs) sites were selected. An evolutionary tree was constructed using these SNPs through the software IQ-TREE-2.2.0 [36].

### 4.3. BSA Analysis and Initial Mapping

Fresh leaves from the melon plants (P_1_, P_2_, F_1_, F_2_) were collected two weeks after planting and stored at −80 °C. DNA was extracted using a modified CTAB protocol [37]. For BSA-seq [38], DNA from 18 multi-CN, 18 normal-CN F_2_ plants, and P_1_-CR465 was sequenced by Annoroad Gene Technology with a target coverage of 20×. The analysis was conducted against the DHL92 V4.0 melon reference genome http://cucurbitgenomics.org/v2/organism/23 (accessed on 2 March 2023). After performance quality control using FastQC-0.12.1 [39], the data exhibited a high quality with an average Q30 of 90.91% and a mean GC content of 36.37%. The normal-CN pool, multi-CN pool, and P_1_-CR465 generated 22, 18.4, and 18.4 Gb of raw data, respectively, with genome mapping rates of 95.83%, 97.89%, and 98.36% and average sequencing depths of 32.44×, 27.09×, and 27.17×, respectively. SNP/INDEL variants were identified using BWA-0.7.17, SamTools-1.18, and GATK-4.1.4.0. Significant associations with carpel traits were determined using a 2.2 Mb sliding window and a 10 Kb step [38,40,41,42], with a 95% confidence threshold.

### 4.4. Fine-Mapping

Genotyping was performed on the CR465 × W336 F_2_ population, targeting the candidate region identified by BSA-seq. KASP markers were developed from variants with ΔSNP values above 0.6 and parent read depths over 10. Primer designs were facilitated by Primer3 Plus (http://www.primer3plus.com/index.html) accessed on 6 May 2023, with FAM and HEX labels on the forward primers, listed in Supplementary Table S1. The markers were synthesized by Qingke Biological Company and genotyping was executed using the high-throughput Gene Matrix system at Qingdao Agricultural University, covering the parental, F_1_, and F_2_ groups.

### 4.5. Expression Analysis and Prediction of Protein Structures

Collect new shoot apices of lateral branches from plants, taking 1–3 per plant, with fifteen plants from each of the two extreme pools. Repeat the sampling for three consecutive days using the same method. Place samples of the same genotype in the same centrifuge tube for collection, rapidly freeze in liquid nitrogen, and store at −80 °C for RNA extraction. RNA extraction was performed with the Huayueyang Plant RNA Kit, gene expression levels were quantified using qRT-PCR, cDNA was synthesized with the Promega (A2790) kit, and SYBR Green Master (Toray) was used for qRT-PCR. Primers were designed using Primer Premier 5 based on the melon genome DHL92 v4.0 and synthesized by Beijing Tsingke Biotech (Beijing, China), as detailed in Supplementary Table S2. relative expression calculated by the 2^−ΔΔCT^ method. Protein structure prediction was performed using the online version of AlphaFold2, with the results visualized using PyMOL-2.5.5. The website is (https://colab.research.google.com/github/deepmind/alphafold/blob/main/notebooks/AlphaFold.ipynb) accessed on 18 November 2023.

## 5. Conclusions

In this study, we first screened and identified a multi-CN *melo* material. Phenotypic observation revealed that the CNs had already differentiated before flowering. Genetic analysis of this material showed that the CNs in *melo* are controlled by a single gene, and the locus controlling this trait was mapped to a 1.8 M region on chromosome 12. Through a cis-trans test, the candidate gene was identified as *CmCLV3*. By comparing with *agrestis*, it was found that the variation in the CNs might be caused by different mutations in *CmCLV3*.

## Figures and Tables

**Table 1:** Separation and analysis of CN in *melon.*

Generations	Plants	Normal-CN (Mean)	Multi-CN (Mean)	Expected Ratio	Actual Ratio	χ2	*p* Value
W336(P_1_)	14	14 (3.05)	0				
CR465(P_2_)	33	0	33 (4.49)				
F_1_	17	17 (3.33)	0				
F_2_-2022	178	142 (3.18)	41 (4.31)	3:1	3.46:1	0.667	0.412

**Table 2:** The correspondence between genotype and phenotype in the results of the cis-trans test.

Sample Name	*CmCLV3^H1^*	*CmCLV3^H2^*	Carpel Number (Mean)
A-F_1_ × ivf05 F_1_-1	C/G	A/A	normal
A-F_1_ × ivf05 F_1_-2	C/G	A/T	multi
A-F_1_ × ivf05 F_1_-3	C/G	A/T	multi
A-F_1_ × ivf05 F_1_-4	C/G	A/T	multi
A-F_1_ × ivf05 F_1_-5	C/G	A/T	multi
A-F_1_ × ivf05 F_1_-6	C/G	A/T	multi
A-F_1_ × ivf05 F_1_-7	C/G	A/T	multi
B-F_1_ × ivf05 F_1_-1	C/G	A/A	normal
B-F_1_ × ivf05 F_1_-2	C/G	A/T	multi
B-F_1_ × ivf05 F_1_-3	C/G	A/A	normal
B-F_1_ × ivf05 F_1_-4	C/G	A/T	multi
B-F_1_ × ivf05 F_1_-5	C/G	A/T	multi
B-F_1_ × ivf05 F_1_-6	C/G	A/T	multi
B-F_1_ × ivf05 F_1_-7	C/G	A/A	normal
B-F_1_ × ivf05 F_1_-8	C/G	A/T	multi
B-F_1_ × ivf05 F_1_-9	C/G	A/A	normal
B-F_1_ × ivf05 F_1_-10	C/G	A/A	normal

## Data Availability

All data are available within the article and the Supplementary Materials. All constructs are available upon request.

## Supplementary Materials

The supporting information can be downloaded at https://www.mdpi.com/article/10.3390/ijms25116011/s1.
